# Stroke Care in Young Patients

**DOI:** 10.1155/2013/715380

**Published:** 2013-02-26

**Authors:** L. Tancredi, F. Martinelli Boneschi, M. Braga, I. Santilli, C. Scaccabarozzi, P. Lattuada, M. Sessa, L. Fumagalli, S. Iurlaro, I. Neromante, M. L. De Lodovici, D. V. Roccatagliata, G. Giacalone, M. Arnaboldi, V. Crespi, E. Agostoni, G. C. Comi, C. Ferrarese, R. Sterzi

**Affiliations:** ^1^Stroke Unit, Neurology Department, Azienda Ospedaliera Sant'Anna Hospital, Via Ravona 1, 22020 San Fermo della Battaglia, Como, Italy; ^2^Institute of Experimental Neurology (INSPE) and Stroke Unit, San Raffaele Scientific Institute, 20132 Milano, Italy; ^3^Stroke Unit, Neurology Department, Azienda Ospedaliera di Desio e Vimercate, 20871 Vimercate, Italy; ^4^Stroke Unit, Neurology Department, Azienda Ospedaliera Niguarda Cà Granda, 20162 Milan, Italy; ^5^Stroke Unit, Neurology Department, Azienda Ospedaliera A. Manzoni, 23900 Lecco, Italy; ^6^Stroke Unit, Neurology Department, Azienda Ospedaliera Ospedale San Carlo Borromeo, 20153 Milan, Italy; ^7^Stroke Unit, Neurology Department, Azienda Ospedaliera San Gerardo, 20900 Monza, Italy; ^8^Neurology Department, Azienda Ospedaliera di Lodi, 26900 Lodi, Italy; ^9^Neurology Department, Azienda Ospedaliera di Busto Arsizio, 21052 Varese, Italy; ^10^Neurology Department, Ospedale di Circolo e Fondazione Macchi, 21100 Varese, Italy

## Abstract

The aims of this study were (i) to evaluate the clinical features of a consecutive series of young patients with ischemic stroke and (ii) to assess the changes in the clinical management of these patients over the study period. All consecutive cases of young adults aged 16 to 44 years, with ischemic stroke, that were admitted between 2000 and 2005 in 10 Italian hospitals were included. We retrospectively identified 324 patients. One or more vascular risk factors were present in 71.5% of the patients. With respect to the diagnostic process, an increase in the frequency of cerebral noninvasive angiographic studies and a decrease in the use of digital subtraction angiography were observed (*P* < 0.001 and *P* = 0.03, resp.). Undetermined causes decreased over 5-year period of study (*P* < 0.001). The diagnosis of cardioembolism increased. Thrombolysis was performed for 7.7% of the patients. PFO closure (8%) was the most frequently employed surgical procedure. In conclusion, the clinical care that is given to young patients with ischemic stroke changed over the study period. In particular, we detected an evolution in the diagnostic process and a reduction in the number of undetermined cases.

## 1. Introduction

In Western countries, approximately 5.5% of all strokes occur in patients who are younger than 45 years of age [1], and the occurrence of ischemic stroke in young adults is 10.8 per 100,000 per year [2]. Despite the lower mortality rate of cerebral ischemia in the young, there is a high social impact of this disease [3, 4]. Recent years have seen remarkable progress in basic and clinical stroke research, and these advances have resulted in improved clinical care and diagnostic workups, as well as a better understanding of stroke pathogenesis [5]. The most important developments in this respect are represented by the increased attention that is placed on detecting cardiac and coagulation defects, screening for novel risk factors [6], improving advanced noninvasive brain imaging strategies, and using intravenous thrombolysis during acute ischemic stroke [5]. 

Although there are many published reports concerning stroke in young adults [2–4, 7], only a small number of these studies considers the evolution of the clinical management of young patients with ischemic stroke [2, 8].

The present work is a multicentric retrospective study that included all consecutive patients aged 16 to 44 years with ischemic stroke who were admitted to 10 Northern Italian hospitals. The objectives of the study were (i) to analyze the clinical features of this population and (ii) to examine the changes in patient clinical care over the study period, including changes in the diagnostic process and patient treatment that occurred over the study period.

## 2. Patients and Methods

All patients aged between 16 and 44 years with acute ischemic stroke who were admitted to 10 hospitals in the Lombardy region, the most populated of Italy, between January 2000 and December 2005 were included in this study. The clinical centers were categorized as follows: seven were equipped with a stroke unit [10], and the remaining three had a dedicated stroke team but no stroke unit. Furthermore, all of the centers were homogeneous with regard to the availability of intensive care, neurosurgery, vascular surgery, neuroradiology, cardiology, and early rehabilitation. 

Acute ischemic stroke was defined in accordance with the Cooperative Young Stroke Study Criteria [4]. The exclusion criteria were as follows: ischemic stroke due to complications of other intracranial diseases, such as subarachnoid hemorrhage, sinus venous thrombosis, severe head trauma, or transient ischemic attack (TIA).

We constructed our patient dataset by searching each hospital's database. The medical records of all consecutive patients aged between 16 to 44 years with ICD 9 cm codes that were between 433.00 and 434.91 were retrospectively reviewed by a team of stroke neurologists to determine if the cases met the definition of ischemic stroke. All of the clinical departments of each hospital, except pediatrics (which admit patients who are younger than 16 years of age), were involved. The data were collected according to a predefined protocol. 

All of the following data were registered: demographic characteristics, family history, risk factors [11], neurological examination findings, diagnostic data, and the type of treatment. The evaluated stroke risk factors and applied definitions are shown in the supplemental Appendix (see Appendix: Details of registered cerebrovascular risk factors and their definitions in Supplementary Material available online at http://dx.doi.org/10.1155/2013/715380). We retrospectively applied consistent definitions for risk factors over the study period. The stroke subtypes were classified according to the following Bamford criteria [12]: lacunar infarct (LACI), partial anterior circulation infarct (PACI), total anterior circulation infarct (TACI), and posterior circulation infarct (POCI). The stroke etiology was classified according to the following TOAST criteria [13]: large-vessel disease (LVD), small-vessel disease (SVD), cardioembolic stroke (CE), other determined cause (OTH), and undetermined cause (UND). 

The time of delay from the stroke onset to the hospital arrival, mortality, and clinical complications during hospitalization were also registered. 

The study's patient sample was arbitrarily divided into the following three age groups for further comparisons with a previous report of young stroke in Italian population [7]: 16–29 years of age (Group 1), 30–39 years of age (Group 2), and 40–44 years of age (Group 3).

The statistical analyses were performed using SPSS statistical software (version 10.1). The chi-squared analysis, Student's *t*-tests, and one-way analysis of variance were used to compare the categorical and continuous variables, respectively. The relationships between the variables were assessed using correlation and regression analyses. The univariate and multivariate logistic regression analyses, which were adjusted for hospital and gender, were performed to test the impact of age at onset of ischemic stroke on the risk of exhibiting the following common risk factors (present in >5% of the population): obesity, hypertension, hypercholesterolemia, and migraine. Additional multivariate models, which were adjusted for hospital and gender, tested the impact of age at onset of stroke on the classification (Bamford) and etiology of the stroke (TOAST). Lastly, a multivariate approach, which was adjusted for hospital and gender, tested the role of stroke subtypes and age at onset of ischemic stroke on the likelihood of returning home following the clinical event.

The strength of the association between the predictors and the dependent variable was assessed using the means of the odds ratios (OR) and relative 95% confidence intervals (95% CI).

## 3. Results

Three hundred and twenty-four subjects who were aged between 16 and 44 years and who were affected with ischemic stroke between January 2000 and December 2005 were included in the study. The mean age at onset was 36.7 years, with 162 women and 162 men. This sample represents 2.7% of the total number of ischemic strokes in patients aged 16 to 80 years who were registered in the study period in the Lombardy region (324/12108) [14].

In total, 303 patients (93.5%) were admitted to the neurological ward, and the remaining patients were admitted to other wards (cardiology, internal medicine, neurosurgery, or intensive care units). 

No significant differences were observed in the demographic data between the centers with respect to gender or the age of onset distributions.

The baseline characteristics and the risk factor frequencies, stratified by the age groups, are reported in Table 1. Among the risk factors with frequencies that were greater than 5% (Table 1), one or more were present in 71.5% of the patients (33.3% of the patients had one risk factor, 22.8% of the patients had two risk factors, and 15.4% of the patients had three or more risk factors). No risk factor was present in 28.5% of the patients. 

Multivariate analyses, which were adjusted for hospital and gender, indicated that hypercholesterolemia is more frequent in older patients (Group 1 versus Group 3: OR 0.97, 95% CI 0.53–1.80, *P* = 0.04), obesity is more frequent in older patients (Group 2 versus Group 3: OR 0.22, 95% CI 0.07–0.75, *P* = 0.01), and migraine is more frequent in younger patients (Group 2 versus Group 3: OR 2.30, 95% CI 1.13–4.70, *P* = 0.02).

Hypertension was present in 50% of the patients with LVD, in 56.9% of those with SVD, in 38.7% of those with CE, in 36.2% of those with OTH, and in 32.2% of those with UND. Smoking was present in 33.3% of the patients with LVD, in 52.9% of those with SVD, in 8.1% of those with CE, in 18.1% of those with OTH, and in 10.3% of those with UND. Hypercholesterolemia was present in 16.7% of the patients with LVD, in 31.4% of those with SVD, in 21% of those with CE, in 14.9% of those with OTH, and in 19.5% of those with UND. The frequency of hypertension was higher in the LVD than in CE (*P* = 0.02), OTH (*P* < 0.001) and UND (*P* = 0.003) categories. Hypertension was also more prevalent in patients with SVD than in those with CE (*P* < 0.001) or OTH (*P* < 0.001). The prevalence of smoking was higher in patients with LVD or SVD; however, the difference was significant only for SVD when compared to OTH (*P* = 0.02) and UND (*P* = 0.004). Hypercholesterolemia was present in all of the stroke subtypes, without any significant differences between them.

The time delay from stroke onset to hospital arrival was <3 hours for 107 patients (33.0%), between 3 and 6 hours for 67 patients (20.7%), between 6 and 24 hours for 68 patients (21.0%), and greater than 24 hours for 63 patients (19.4%). The time delay was unknown for 19 patients (5.9%). No significant changes of time delay over 5-year period of study were observed. 

For the diagnostic workup, EKGs and brain CT scans were acquired for all of the patients. Brain MRIs were acquired in 237 patients (73.1% of the total). All of the patients underwent a minimum of one extracranial circulation evaluation: an extracranial duplex ultrasonography was performed in 238 patients (73.4%), an extracranial MR angiography was performed in 67 patients (20.7%), and an extracranial CT angiography was performed in 27 patients (8.3%). Intracranial MR angiography was performed in 118 patients (36.4%), intracranial CT angiography was performed in 19 patients (5.9%), and digital subtraction angiography was performed in 102 patients (31.5%). Cardiologic assessments included transthoracic echocardiography (192 patients, 59.3%), transesophageal echocardiography (146 patients, 45.1%), transthoracic echocardiography with contrast injection (76 patients, 23.5%), and transcranial Doppler ultrasonography (32 patients, 9.9%). 

**Figure 1:**
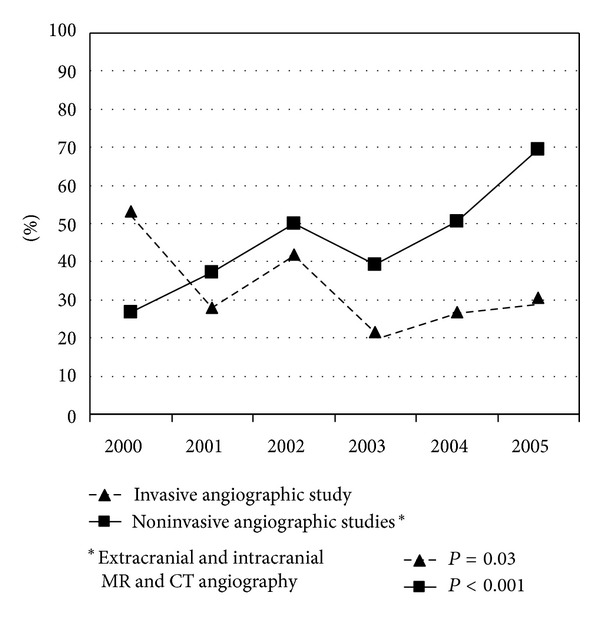
Changes over time (2000–2005) of the frequency of angiographic studies.

The type of diagnostic workup that was performed changed over the study period. When we performed the analysis over the 5-year period, the proportion of the patients who underwent a brain MRI progressively increased from 60% (2000) to 80.6% (2005), *P* = 0.1. The frequency of the patients who underwent a minimum of one noninvasive angiographic study of the cerebral circulation (extracranial and intracranial MR angiography, extracranial and intracranial CT angiography) increased: 26.7% (2000), 37.2% (2001), 50% (2002), 39.2% (2003), 50.7% (2004), 69.4% (2005), *P* < 0.001. A reduction in the use of digital angiography was observed: 53.3% (2000), 27.9% (2001), 42% (2002), 21.6% (2003), 26.7% (2004), 30.6% (2005), *P* = 0.03, Figure 1. The proportion of the patients who underwent a minimum of one study to detect a PFO increased from 56.7% (2000) to 66.7% (2005), *P* = 0.08. 

Coagulation testing (i.e., testing for the presence of antiphospholipid antibodies, protein C, protein S, antithrombin III, factor V Leiden, and prothrombin gene analyses and homocysteine plasma levels) was performed in 271 patients (83.6% of the patients). The findings of these tests were abnormal in at least one test in 84 patients (30.9%). In 19/324 patients (5.9%), thrombophilia was recognized as the underlying cause of stroke: antiphospholipid syndrome was present in ten patients, mutations in the factor V Leiden or prothrombin genes were detected in four patients, a deficiency of protein S was observed in one patient, and hyperhomocysteinemia (persistently elevated plasma levels >20 *μ*mol/L measured at admission and after three months) was observed in four patients.

With respect to the stroke type classification, LACI was diagnosed in 44 patients (13.6%), TACI was diagnosed in 35 patients (10.8%), PACI was diagnosed in 130 patients (40.1%), and POCI was diagnosed in 115 (35.5%). No significant changes over the study period were observed in the relative percentages of these diagnoses (*P* = 0.08). 

Multivariate analyses, which were adjusted for age and center, indicated that the patients who were aged between 16 and 29 years (Group 1) exhibited a lower frequency of LACI classification than did older patients (40–44 years of age: OR = 0.42 (95% CI = 0.19–0.91), *P* = 0.03). 

The stroke etiology was classified according to the TOAST criteria: LVD in 30 patients (9.3%), SVD in 51 patients (15.7%), CE in 62 patients (19.1%), UND in 87 (26.9%), and OTH in the remaining 94 patients (29%). 

In the cardioembolic group, high-risk factors were identified in 17 patients (valvular heart disease in 5 patients, dilated cardiomyopathy in 5 patients, atrial fibrillation in 4 patients, myocardial infarction in 2 patients, and congenital cardiac malformation in 1 patient). Low- or uncertain-risk sources were identified in 45 patients (PFO in 32 patients and PFO with atrial septal aneurysm in 13 patients).

The subtypes of uncertain diagnoses included an incomplete evaluation in 23 patients, multiple possible etiologies in 13 patients, and a negative evaluation in 51 patients. 

The category of ischemic stroke as a result of other causes included the following items: arterial dissection (47), thrombophilia (19), systemic lupus erythematosus (7), inflammatory bowel disease (1), drug abuse (5), migrainous infarct (5), vascular malformations (5), hematological disease (2), Sneddon's syndrome (2), and CADASIL (1). 

**Figure 2:**
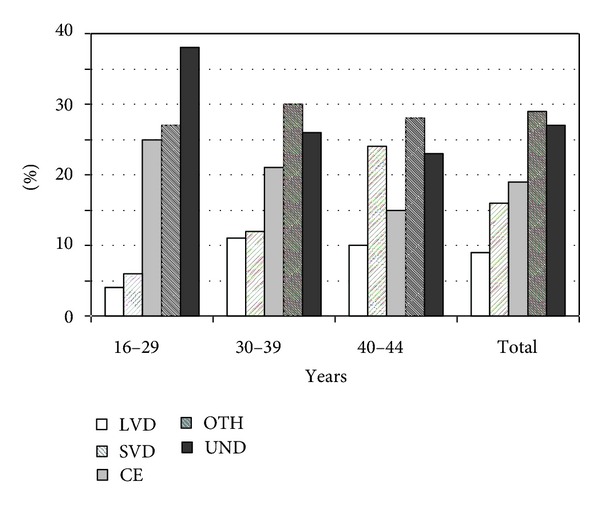
TOAST criteria stratified by age classes.

The stroke etiology was stratified by age, as illustrated in Figure 2. According to the multivariate model, the risk of exhibiting a LVD or SVD etiology versus all of the other causes (i.e., CE, UND and OTH) was significantly lower when the patient was under 30 years of age at onset (*P* = 0.02; OR = 0.21 (95% CI = 0.07–0.56)) when compared to older patients. 

**Figure 3:**
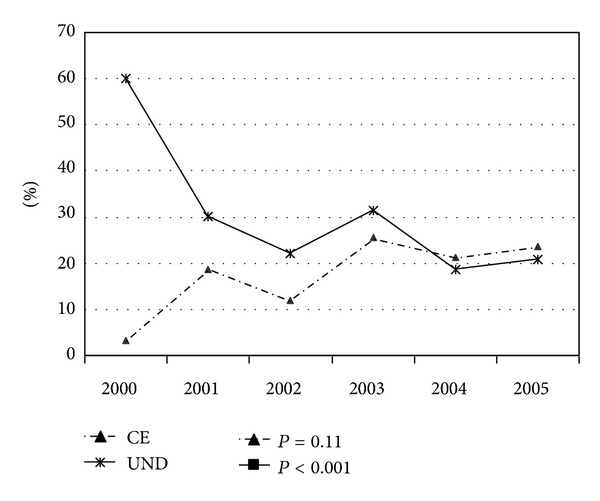
**:** TOAST classification: changes over time (2000–2005) of undetermined and cardioembolic strokes.

Only UND and CE showed changes over time. The proportion of undetermined cases decreased as follows: 60% (2000), 30.2% (2001), 22% (2002), 31.4% (2003), 18.7% (2004), 20.8% (2005), *P* < 0.001. Cardioembolic strokes increased as follows: 3.3% (2000), 18.6% (2001), 12% (2002), 25.5% (2003), 21.3% (2004), 23.6% (2005), *P* = 0.11, Figure 3.

The medical treatments and surgical procedures that were used are listed in Table 2. Percutaneous PFO closure was reserved for cases of PFO that were associated with an atrial septal aneurysm, severe right-left shunting, and/or deep vein thrombosis. The exclusion of other possible causes of ischemia was also required for this procedure to be performed. No significant changes over the study period were observed.

The mortality rate during hospitalization was 2/324 (0.6%). The intrahospital complications included the following: epileptic seizures (6), urinary tract infections (18), respiratory infections (6), fever (12), and depressive symptoms (19). Among the surviving patients, 76.1% returned home, and 23.9% were transferred to a neurorehabilitation department. Multivariate analyses, which were adjusted for age and center, indicated that the probability of returning home was significantly higher in LACI (95.3%) than in non-LACI (73.1%) patients (*P* = 0.02; OR = 11.03 (95% CI = 2.48–49.08)). A higher probability of returning home was also observed in patients who were aged between 16 and 29 years (80.4%) and for those who were between 30 and 40 years of age (78.4%) when compared to the older group (71.9%). These differences, however, did not reach statistical significance (*P* = 0.13 and *P* = 0.08, resp.).

## 4. Discussion

Our study describes the clinical characteristics of 324 Italian patients who were affected with ischemic stroke and who were between 16 and 44 years of age. Here, we evaluate the changes over the study period in the type of clinical care that was given to these patients.

The study sample represents 12.4% of all of the ischemic stroke cases in young adults in the Lombardy region during the study period [14]. Furthermore, in agreement with previous studies [15], the collected sample represents 2.7% of all stroke cases that were admitted to the ten participating hospitals. Most of the patients (93.5%) were admitted to a neurological ward. The standard practice in Italy is to treat stroke patients, particularly younger patients, in the neurology department.

The sample was homogeneous across the participating centers, with no major differences in age or sex. As expected, there was a general increase in the risk of the disease with age [2, 7]. We did not observe a significant prevalence of female patients who were younger than 29 years of age, which is similar to what has been reported in other studies [2, 7].

As is shown in Table 1, modifiable risk factors (particularly smoking and hypertension) are strongly represented. This relationship was observed to be stronger than was reported in earlier studies [16] but was similar to more recent findings [2, 7]. A higher frequency of hypertension, hypercholesterolemia, and obesity was observed in the older age group (40 to 44 years of age). Other observations included (i) the higher prevalence of hypertension in the LVD and SVD category and (ii) the higher prevalence of smoking in the SVD category. Furthermore, 14.8% of the patient sample had experienced previous vascular events, including ischemic stroke and TIA. 

Moreover, a high proportion of the patients had previously suffered from symptomatic vascular disease. In conclusion, conventional vascular risk factors are not only important in older patients with ischemic stroke, but also in this younger population under the age of 44. 

Although a history of migraine was present in approximately 20% of the patients who were younger than 39 years of age, only 5 patients (1.5%) fulfilled the criteria for a probable migraine-induced stroke [9]. The role of migraine in stroke is controversial. Our data contrast with those of most previous reports, which state a 3%–20% prevalence of migrainous stroke [17] but are similar to more recent findings (<1%) [2].

The topography of cerebral infarction appears to confirm the recently described prevalence of posterior circulation involvement in young patients [2, 7]. The proportion of infarcts in the posterior territory varies from 25% to 46% in patients who are under 45 years of age which is significantly higher than what is observed for older patients (11.9%) [18]. The high proportion of vertebrobasilar stroke that was observed in the present study (35.5% of all of the patients) may be attributable to the frequent use of MRI, which is more useful for the analysis of posterior circulation. Arterial dissections involving posterior circulation are also frequent in young adults. Furthermore the young might have distinctive underlying pathophysiological mechanisms compared with the elderly. 

The relationship between the TOAST classification and the age groups has rarely been detailed [7]. LVD and SVD are underrepresented causes of stroke in patients who are younger than 39 years of age. The prevalence of a vascular cause of stroke increases with age, primarily due to the presence of atherosclerosis in older patients [7, 19].

Additionally, the proportion of cases that are classified as resulting from other causes was similar to that reported in other studies [3].

The second aim of the study was to describe the modification of both the clinical care and the diagnostic and therapeutic workup for the studied patients throughout the study period (2000–2005).

Only a small number of reports have analyzed changes in the etiological diagnosis of ischemic stroke in young adults over time [2, 8]. In the five-year span of our study, there was a significant reduction in the proportion of undetermined causes based on the TOAST classification. This finding was paralleled by an increase in size of the cardioembolic group. This could be attributable to the major impact that mild cardiac defects, such as PFO and atrial septal aneurysms, can have in stroke pathogenesis in the young compared with the elderly. However, it is necessary to be cautious in interpreting and handling a diagnosis of PFO [20]. First, it is occasionally difficult to detect paradoxical emboli, in situ thrombosis, and transient atrial arrhythmias. Second, other causes of stroke must be carefully excluded. Third, PFO detection can be an incidental finding. Additionally, the systematic application of a neuroradiological diagnostic workup (including brain MRIs and the analysis of extra- and intracranial circulation) could aid in the identification of other causes. A similar evolution in etiological diagnoses was recently reported by Varona et al. [8]. 

Another change of the diagnostic approach was the increased prevalence of noninvasive angiographic studies. It was counterbalanced by a reduction in the use of digital angiography. Although the use of traditional digital angiography is decreasing, it is still used rather frequently (in 30.6% of the cases in this study), principally to identify otherwise unknown causes of stroke. 

Despite the observed changes in the types of diagnoses that were given over the study period, undetermined cases remain frequent. In our opinion, the prevalence of certain diagnoses (e.g., neurocutaneous syndromes, Fabry's disease and other monogenic disease, arterial vasospasm, and illicit drug use) may have been underestimated given that such causes were not overtly considered by local protocols during the study period. Long-term followup of young patients with ischemic stroke could help to identify rare causes of disease. As previously reported [2, 7], coagulation studies can detect a possible cause of stroke in only a minority of patients. 

With respect to the therapeutic approaches that were adopted, 4% of the patients underwent intravenous thrombolysis, and 3.7% underwent intra-arterial thrombolysis. Therefore, as was observed in older patients [21], only a minority (7.7%) of the patients who arrived within the 6-hour therapeutic window (53.7%) underwent thrombolytic treatment. This proportion did not change over the study period despite the recently reported increase in awareness of the efficacy of thrombolytic treatment in young adults [22–24]. Wagner and Lutsep [21] suggest that poor disease awareness is responsible for the limited administration of thrombolysis in young people.

The most frequent surgical intervention was percutaneous PFO closure (8%). This practice reflects the emerging role of septal abnormalities in young stroke patients over the study period. Carotid surgery is restricted to a minority of young patients. 

Intrahospital mortality and complications were rare in this study. The short period of observation that corresponded to the acute phase and the young age of the patient sample may explain the low mortality rate that was observed. Short-term outcomes were generally good, particularly for lacunar infarcts. However, the prognostic implications of cerebral small vessel disease are still unclear, as is reported in older patients [25]. 

The primary limitation of this study is the retrospective design. The advantages include the exclusion of nonischemic stroke and TIA patients, the large number of the patients in the sample, and the relatively short study period. The multicentric setting of the study does not appear to have influenced our results, as no major differences were identified between the centers.

In conclusion, the present study describes the characteristics, the diagnostic flow charts, and the treatment strategies for ischemic stroke in young patients that are currently used in clinical practice. Our results highlight the importance of vascular risk factors in young adults and suggest the requirement for stroke prevention strategy optimization in this population. The improvement in diagnostic workup over the study period reduced the number of undetermined strokes. Certain limitations remain in the management of these patients, especially with regard to the low impact of thrombolysis. The relatively favorable short-term prognosis that was observed in the study sample should be reassessed in a longer follow-up study.

## Supplementary Material

Details of registered cerebrovascular risk factors and their definitions.

## Figures and Tables

**Table 1:** Baseline characteristics and risk factors stratified by age classes.

Age classes	16–29 years	30–39 years	40–44 years	Total
Patient number (%)	52 (16.0)	141 (43.5)	131 (40.4)	324 (100)
Male/female	27/25	71/70	64/67	162/162

Risk factors (%)				

Smoking	17 (32.7)	51 (36.2)	62 (47.3)	130 (40.1)
Hypertension	2 (3.8*)	25 (17.7*)	41 (31.3)	68 (21)
Oral contraceptives	12 (23.1)	29 (20.6)	27 (20.6)	68 (21°)
Hypercholesterolemia	5 (9.6^†^)	29 (20.6)	31 (23.7)	65 (20.1)
Migraine	11 (21.2)	29 (20.6^†^)	15 (11.5)	55 (17)
Hyperhomocysteinemia	10 (19.2)	23 (16.3)	18 (13.7)	51 (15.7)
Previous vascular events	7 (13.5)	20 (14.2)	21 (16)	48 (14.8)
Family history of stroke	2 (3.8)	10 (7.1)	13 (9.9)	25 (7.7)
Obesity	2 (3.8)	4 (2.8^†^)	13 (9.9)	19 (5.9)
Others°°				

^†^ *P* < 0.05 *P < 0.01.

°When tested in women is 42% of the sample.

°°Other risk factors with frequency below 5% were trauma 12 (3.7%); diabetes mellitus 11 (3.4%); heavy drinking 10 (3.1%); illicit drug use 5 (1.5%); ischemic cardiopathy 5 (1.5%); atrial fibrillation 4 (1.2%); pregnancy/puerperium 6 (1.9%).

**Table 2:** Therapeutic approach in young patients with ischemic stroke.

Treatment	*n* (%)
Antiplatelet therapy	238 (73.5)
Anticoagulant therapy	70 (21.6)
Hypertension treatment	60 (18.5)
Vitamin therapy	46 (14.2)
Patent foramen ovale closure	27 (8.0)
Intravenous thrombolysis	13 (4.0)
Intra-arterial thrombolysis	12 (3.7)
Carotid stenting	2 (0.6)
Thromboendarterectomy	1 (0.3)
Basilar stenting	1 (0.3)
