# Supplementary material for: Stroke Care in Young Patients
Source: Stroke Res Treat. 2013 Feb 26;2013:715380. doi: 10.1155/2013/715380 (PMC3600297; doi:10.1155/2013/715380)
Supplement: Supplementary file 1 — Details of registered cerebrovascular risk factors and their definitions. [file 715380.f1.doc]

**Appendix. Details of registered cerebrovascular risk factors and their d**efinitions

| **Risk factors (in alphabetical order)** | |
| --- | --- |
| ***Risk factor*** | ***Definition*** |
| Atrial fibrillation | Presently diagnosed or history of chronic/paroxysmal atrial fibrillation |
| Diabetes mellitus, type 1 and 2 | Treated or defined according to the 1999 World Health Organization criteria as fasting plasma glucose ≥7.0 mmol/L (126 mg/dL) |
| Family history of stroke | History of ischemic/hemorrhagic stroke or TIA in a first-degree relative |
| Heavy drinking | More than clearly moderate drinking, *i.e.*, estimated intake of >200g of pure alcohol per week constantly as disclosed by the patient or a relative |
| Hypercholesterolemia | Treated or total cholesterol level ≥5.2 mmol/L (200 mg/dL), low-density lipoprotein level ≥3.0 mmol/L (116 mg/dL), or high-density lipoprotein level <1.0 mmol/L (39 mg/dL) |
| Hyperhomocysteinemia | Persistently elevated plasma levels > 20 μmol/L |
| Hypertension | Treated or a history of hypertension according to the 2003 World Health Organization criteria as systolic blood pressure ≥140 mm Hg and/or diastolic blood pressure ≥90 mm Hg on 2 different occasions. |
| Illicit drug use | Within the month prior to stroke. A general question was asked to every patient screened (“have you ever used illegal drugs?”) and in case of positive answer or in case of strong suspicion urine test were performed. |
| Ischemic cardiopathy | Coronary heart disease and myocardial infarction |
| Migraine | Migraine was defined according to the International Headache Society criteria [16] |
| Obesity | Body-mass index ≥30 or patient clearly stated as heavily obese |
| Oral contraceptives | Under oral contraceptive therapy at stroke onset |
| Pregnancy/puerpuerium | Ongoing pregnancy/The approximate six-week period lasting from childbirth to the return of normal uterine size |
| Previous vascular events | Any of acute stroke or TIA before the admission |
| Smoking | Smoking constantly ≥1 cigarettes per day within the year prior to stroke |
| Trauma | Blunt head trauma, mechanical trauma to the neck, including neck manipulation |
